# Genome on the move: emergence of hybrid atypical enteropathogenic/enteroaggregative *Escherichia coli* (aEPEC/EAEC) during a diarrheal outbreak in Brazil

**DOI:** 10.1128/spectrum.02774-25

**Published:** 2026-04-03

**Authors:** Daiany R. P. de Lira, Iranildo A. Fernandes, Henrique Orsi, Vincent L. Viala, Luís F. dos Santos, Tânia A. T. Gomes, Waldir P. Elias, Eneas Carvalho, Rodrigo T. Hernandes

**Affiliations:** 1 Instituto de Biociências, Universidade Estadual Paulista (UNESP) 154010 , Botucatu, São Paulo, Brazil; 2 Laboratório de Bioquímica, Instituto Butantan 196591, São Paulo, São Paulo, Brazil; 3 Centro de Bacteriologia, Instituto Adolfo Lutz 89119, São Paulo, São Paulo, Brazil; 4 Departamento de Microbiologia, Imunologia e Parasitologia, Escola Paulista de Medicina, Universidade Federal de São Paulo (EPM - UNIFESP) 544243 , São Paulo, São Paulo, Brazil; 5 Laboratório de Bacteriologia, Instituto Butantan 196591, São Paulo, São Paulo, Brazil; University of Valencia, Paterna, Valencia, Spain

**Keywords:** hybrid aEPEC/EAEC, diarrheal outbreak, genomic plasticity

## Abstract

The plasticity of the *Escherichia coli* genome has allowed the emergence of pathogenic strains with unexpected genetic profiles. During an investigation of a diarrheal outbreak in Brazil, we identified one atypical enteropathogenic (aEPEC), four enteroaggregative (EAEC), and three hybrid aEPEC/EAEC *E. coli* strains, all belonging to the serotype O3:H2. Short-read sequencing and long-read sequencing of these strains were performed to generate draft and complete genome assemblies, which were subjected to comprehensive analyses. The outbreak-associated O3:H2 strains were classified within phylogroup A, assigned to the sequence types ST10 (2 EAEC) or ST8087 (1 aEPEC, 2 EAEC, and 3 hybrid aEPEC/EAEC), and were closely related according to the phylogenetic analysis performed. The comparison of their chromosomes revealed key genetic features in the hybrid aEPEC/EAEC strain, including a prophage carrying genes encoding 3 EPEC-translocated effectors (*nleB2*, *nleF*, and *nleH2*) and the locus of enterocyte effacement (LEE region) subtype 8, identical to that found in the aEPEC strain. Additionally, the plasmid of aggregative adherence (pAA) identified in the EAEC and hybrid aEPEC/EAEC strains shared approximately 100% nucleotide identity across at least 85.7% of their sequences and contained the *aggDCBA* operon and its regulator *aggR*. In conclusion, our findings suggest that all *E. coli* strains of serotype O3:H2 belonging to the ST8087 studied likely originated from a common ancestor, which, through multiple horizontal gene transfer events, contributed to the emergence of the aEPEC and EAEC pathotypes, as well as the hybrid aEPEC/EAEC strain.

**IMPORTANCE:**

This study provides evidence that the high genomic plasticity of *Escherichia coli* has played a key role in the emergence of diarrheagenic strains harboring virulence markers from atypical enteropathogenic (aEPEC) and enteroaggregative (EAEC) *E. coli*, as well as strains with markers from both pathogenic groups, combined in hybrid aEPEC/EAEC strains. Phylogenetic analysis suggests that these strains share a common ancestral lineage within the ST10, from which a branch subsequently differentiated into the ST8087. The presence of mobile genetic elements shared among all strains, alongside others that are pathotype-specific, highlights the mosaic architecture of these genomes. Elucidating this evolutionary process, particularly the generation of *E. coli* strains with novel combinations of virulence genes, is essential for advancing our understanding of the evolution of the diarrheagenic *E. coli* (DEC) genome and its implications for pathogenicity.

## INTRODUCTION

The diversity of virulence strategies used by diarrheagenic *Escherichia coli* (DEC) to colonize and cause disease in the human gastrointestinal tract has led to the classification of DEC into six distinct pathotypes: enteropathogenic *E. coli* (EPEC), enteroaggregative *E. coli* (EAEC), Shiga toxin-producing *E. coli* (STEC), enterotoxigenic *E. coli* (ETEC), enteroinvasive *E. coli* (EIEC), and diffusely adherent *E. coli* (DAEC) (1–3). Furthermore, the EPEC pathotype can be divided into two subgroups: typical (tEPEC) and atypical (aEPEC), based on the presence of a high-molecular weight plasmid, known as EPEC adherence factor (pEAF), which is found only in the former group (4–6). Notably, the pEAF contains a set of 14 genes responsible for encoding proteins involved in the biogenesis of a type IV fimbriae, known as bundle-forming pili (BFP) (7, 8). BFP mediates the formation of the localized adherence (LA) phenotype, characterized by the development of compact microcolonies on the surface of infected epithelial cells (9).

The hallmark of EPEC infections is the formation of a histopathological lesion known as attaching and effacing (AE), which is characterized by three main features: (i) intimate adherence of the bacteria to the epithelial cell, (ii) microvilli effacement, and (iii) the formation of a pedestal-like structure rich in F-actin and other eukaryotic cytoskeletal elements. All proteins required for AE lesion formation are encoded by a set of 41 chromosomal genes located within a pathogenicity island (PAI) of approximately 35 Kb, referred to as the locus of enterocyte effacement (LEE) region. Among the genes in the LEE region, *eae* is particularly notable as it encodes the adhesin intimin, which is responsible for intimate bacterial adherence to the host cell. The adhesin intimin interacts with another bacterial protein, the translocated intimin receptor (Tir), which serves as an intimin receptor once translocated into the host cell cytoplasm via the type 3 secretion system (T3SS) of EPEC (10–14).

In addition to the seven bacterial effectors (EspB, Tir, EspF, Map, EspG, EspH, and EspZ), encoded by genes located within the LEE region, several other effectors encoded by genes situated in distinct PAIs or prophages also utilize the T3SS to be delivered into host epithelial cells. These effectors are collectively referred to as non-LEE effectors (Nle) and are capable of modulating various functions within the eukaryotic host cell, such as the following: disruption of the cell cycle (Cif), inhibition of protein export from the endoplasmic reticulum (NleA), suppression of proinflammatory signaling (NleB, NleC, NleE, and NleH), and inhibition of apoptosis (NleD, NleF, and NleH) (15–17).

EAEC adheres to HeLa and HEp-2 cells in a characteristic pattern known as aggregative adherence (AA), which is characterized by the arrangement of the bacterial cells on the surfaces of epithelial cells and glass coverslips in a manner that resembles stacked bricks (18). This adherence pattern is primarily mediated by a set of chaperone-usher adhesins known as aggregative adherence fimbriae (AAF), of which five distinct variants have been identified to date (AAF/I to AAF/V), or alternatively, by a type IV pilus termed aggregate-forming pilus (AFP) (19–24). The genes encoding the proteins involved in AAF biogenesis are located on a high-molecular weight plasmid known as pAA, which also carries genes encoding several other virulence factors, including toxins, such as Pet (plasmid-encoded toxin) and EAST-1, the antiaggregation protein dispersin, its associated secretion apparatus, and the transcriptional regulator AggR (25).

Many of the genes encoding virulence factors found in the distinct DEC pathotypes are associated with mobile genetic elements, such as plasmids, prophages, and PAIs, that can be transferred among *E. coli* strains via horizontal gene transfer. The acquisition and stable maintenance of these genetic elements have played a key role in the diversification of the *E. coli* species into several pathogenic groups (2, 26, 27).

Recent advances in understanding the genome of DEC have revealed the existence of strains that transcend the traditional classification by harboring virulence factor-encoding genes from two or more DEC pathotypes within their genome. These strains are collectively referred to in the literature as hybrids (28). The presence of multiple virulence factor-encoding genes in a single *E. coli* strain may contribute to the emergence of more virulent pathogens, as exemplified by the hybrid STEC/EAEC of serotype O104:H4, responsible for a diarrheal outbreak in Germany in 2011, which affected approximately 3,900 individuals, 54 of whom died due to this infection (29–31). In addition to STEC/EAEC, several other hybrids have been reported in the literature, including STEC/ETEC, EPEC/EAEC, EPEC/ETEC, and ETEC/EAEC (28, 32).

Of note, a recent study from our laboratory described a diarrheal outbreak caused by *E. coli* of serotype O3:H2, classified as aEPEC, EAEC, or hybrid aEPEC/EAEC (33). In the present study, we dissected the genomes of eight *E. coli* strains of serotype O3:H2 (one aEPEC, four EAEC, and three aEPEC/EAEC hybrids), obtained during the outbreak investigation, with the objective of identifying the mobile genetic elements involved in the construction of these pathotypes, as well as in the emergence of the hybrid aEPEC/EAEC strain.

## RESULTS

### The outbreak-associated *E. coli* O3:H2 strains analyzed are closely related, belong to phylogroup A, and are classified as ST10 or ST8087

Using the Illumina technology, we first sequenced 8 *E. coli* strains of serotype O3:H2, which were collected during a diarrheal outbreak investigation in Brazil (33). The average genome size of the sequenced *E. coli* strains was 4,900,332 base pairs (bp) (ranging from 4,760,815 to 5,145,052), with an average GC content of 50.6% (ranging from 50.51% to 50.69%) (Table S1).

*In silico* analysis of the draft genomes confirmed that all eight strains belonged to serotype O3:H2. These strains were further classified into phylogroup A and assigned to the sequence types (STs) ST10 (IAL7250 and IAL7251) or ST8087 (IAL7252, IAL7253, IAL7254, IAL7255, IAL7256, and IAL7257). We also analyzed the presence of key virulence factor-encoding genes, confirming that these strains could be classified into the following pathotypes: aEPEC (IAL7254), EAEC (IAL7250, IAL7251, IAL7255, and IAL7257), or hybrid aEPEC/EAEC (IAL7252, IAL7253, and IAL7256), based on the presence of *eae*, *aggR*, or both genes, respectively (Table 1; Table S2). A complete list of all identified virulence factor-encoding genes is provided in Table S2.

**TABLE 1:** Main genomic characteristics of the eight outbreak-associated *E. coli* strains sequenced

*E. coli* strain identification	Diagnostic genetic markers^*a*^	DEC pathotype classification^*b*^	*In silico* analysis		
			Serotype	Phylogroup	Sequence type
IAL7254	*eae*	aEPEC	O3:H2	A	8087
IAL7250	*aggR*	EAEC	O3:H2	A	10
IAL7251	*aggR*	EAEC	O3:H2	A	10
IAL7255	*aggR*	EAEC	O3:H2	A	8087
IAL7257	*aggR*	EAEC	O3:H2	A	8087
IAL7252	*eae*/*aggR*	aEPEC/EAEC	O3:H2	A	8087
IAL7253	*eae*/*aggR*	aEPEC/EAEC	O3:H2	A	8087
IAL7256	*eae*/*aggR*	aEPEC/EAEC	O3:H2	A	8087

^*a*^ As shown in Table S2.

^*b*^ EAEC; enteroaggregative *E. coli*; aEPEC, atypical enteropathogenic *E. coli*.

**Fig 1:**
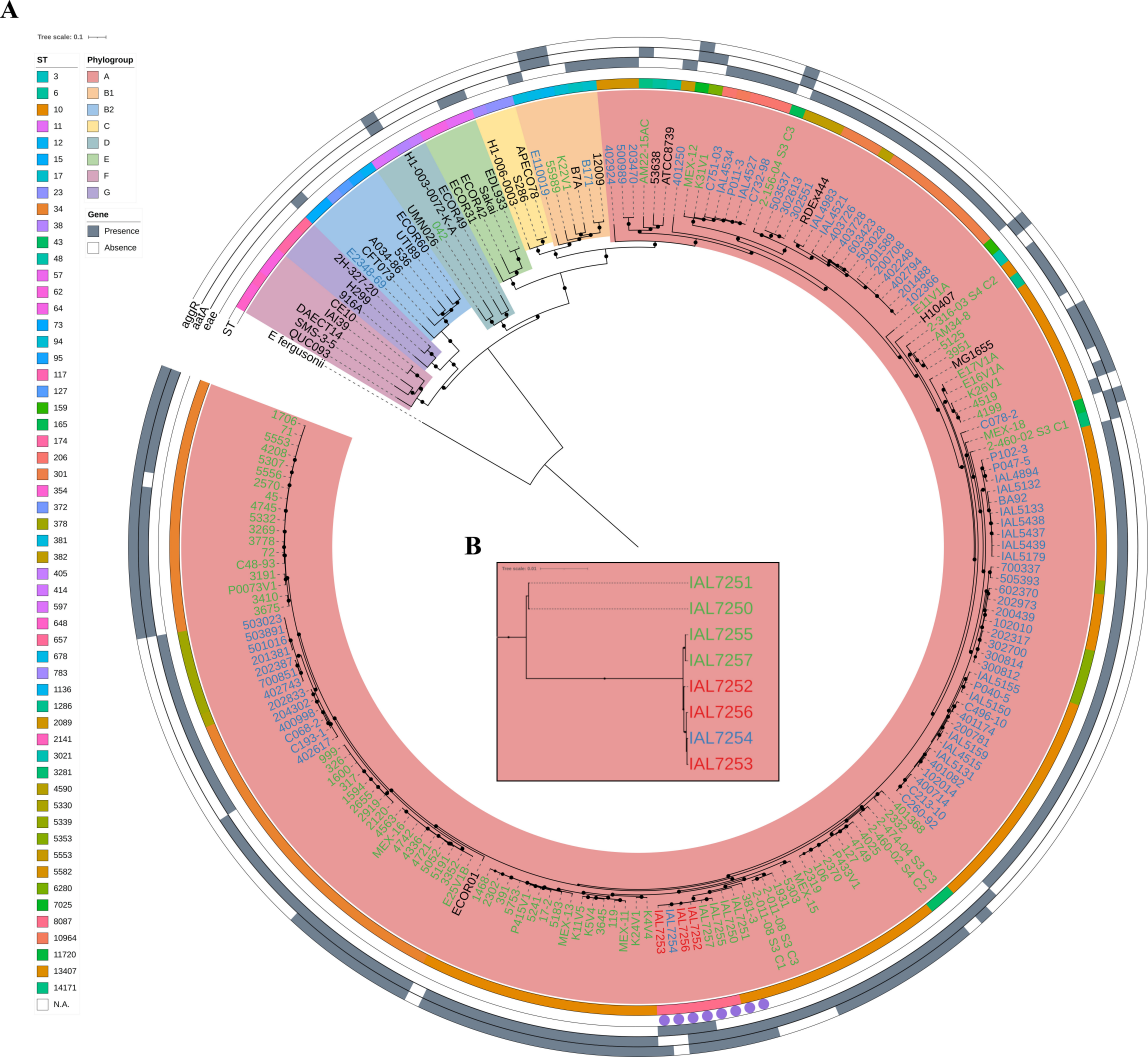
Phylogenetic analysis reveals that outbreak-associated *Escherichia coli* O3:H2 are closely related and cluster with ST10 EAEC strains. (**A**) A maximum-likelihood phylogenetic tree, constructed based on the 882,806 single-nucleotide polymorphisms (SNPs) identified across 202 genomes (Table S15), revealed that the eight outbreak-associated *E. coli* strains of serotype O3:H2 grouped into a single phylogenetic cluster. Additionally, the outbreak-associated O3:H2 strains clustered together with several EAEC strains belonging to the ST10. Nodes with Shimodaira–Hasegawa-like (SH-like) support values ≥0.95 are indicated by black circles. The identification of the *E. coli* strains is color-coded according to their pathotype classification: Green, EAEC; blue, tEPEC and aEPEC; red, hybrid aEPEC/EAEC. Reference *E. coli* strains and *E. fergusonii* are shown in black. Furthermore, the branch colors represent the classification of the strains into the distinct *E. coli* phylogroups (A, B1, B2, C, D, E, F, and G). The first (outermost) ring is color-coded by the ST, while the remaining three rings indicate the presence or absence of the *eae*, *aatA*, and *aggR* genes, as described in the legend next to the figure. Of importance, the eight outbreak-associated *E. coli* strains are highlighted with purple circles. (**B**) The inset at the center of the phylogenetic tree shows a magnified view of the eight outbreak-associated O3:H2 strains.

Phylogenetic analysis revealed a close genetic relationship among the eight outbreak-associated *E. coli* strains from the serotype O3:H2, which formed a single clade divided into two distinct branches in the phylogenetic tree (Fig. 1A). The first branch included one aEPEC (IAL7254), two EAEC (IAL7255 and IAL7257), and three hybrid aEPEC/EAEC (IAL7252, IAL7253, and IAL7256) strains, all belonging to the ST8087. The second branch comprised two EAEC strains (IAL7250 and IAL7251) from the ST10. Additionally, we observed that the outbreak-associated *E. coli* studied clustered with several EAEC strains belonging to the ST10 (Fig. 1B).

### The hybrid aEPEC/EAEC strain was reliably confirmed by complete genome assembly

To better understand the mobile genetic elements driving the diversification of the *E. coli* of serotype O3:H2 strains into three distinct groups (aEPEC, EAEC, and hybrid aEPEC/EAEC), a representative strain of each pathotype was randomly selected for long-read sequencing, hybrid assembly (combining short and long reads), and further genomic analysis. Since the EAEC strains were classified into two distinct STs, ST10 and ST8087 (Fig. 1B), one representative strain from each ST was chosen. Thus, the four selected strains were as follows: IAL7254 (aEPEC: ST8087), IAL7250 (EAEC: ST10), IAL7255 (EAEC: ST8087), and IAL7252 (hybrid aEPEC/EAEC: ST8087).

The hybrid assembly of the four representative genomes resulted in complete circular DNA molecules, which were easily separated into chromosomes and plasmids based on differences in the size, gene content, and comparison with DNA nucleotide sequences found in the National Center for Biotechnology Information (NCBI) database using the BLASTN tool (Table 2; Tables S1 and S3). As expected, the *eae* gene, a genetic marker of the LEE region in EPEC, was found in the chromosomes of both the aEPEC IAL7254 and the hybrid aEPEC/EAEC IAL7252 strains. Moreover, the two EAEC (IAL7250 and IAL7255) and the hybrid aEPEC/EAEC IAL7252 strains contained a high-molecular weight plasmid carrying EAEC-associated genes, including the *aggR* gene, which encodes a key transcriptional regulator in the EAEC pathotype, and the *aggDCBA* operon, encoding the AAF/I fimbriae (Table 2; Table S4). The presence of the *eae* gene on the chromosome and the *aggR* gene on a high-molecular weight plasmid (pAA) in IAL7252 provides strong evidence for classifying this strain as a hybrid aEPEC/EAEC. Additionally, all genes encoding virulence factors or antimicrobial resistance found on the chromosomes or plasmids of the four representative sequenced strains are listed in Tables S4 and S5.

**TABLE 2:** Circular DNA molecules found in the four representative *E. coli* strains of serotype O3:H2 sequenced

DEC pathotype^*a*^	*E. coli* strains	Circular DNA molecules	Size (base pairs)	Diagnostic markers^*c*^		Accession numbers
				eae	aggR	
EAEC	IAL7250	Chromosome	4,710,432	−	−	JBRFVO020000001.1
		Plasmid 1 (pAA)^*b*^	95,100	−	+	JBRFVO020000002.1
		Plasmid 2	4,076	−	−	JBRFVO020000003.1
		Plasmid 3	2,311	−	−	JBRFVO020000004.1
	IAL7255	Chromosome	4,719,887	−	−	JBRFVM020000001.1
		Plasmid 1 (pAA)^*b*^	84,405	−	+	JBRFVM020000002.1
		Plasmid 2	5,569	−	−	JBRFVM020000003.1
		Plasmid 3	2,311	−	−	JBRFVM020000004.1
aEPEC	IAL7254	Chromosome	4,797,584	+	−	CM130001.1
		Plasmid 1	6,440	−	−	JBRYHU010000002.1
		Plasmid 2	2,311	−	−	JBRYHU010000003.1
aEPEC/EAEC	IAL7252	Chromosome	4,793,422	+	−	JBRFVK020000001.1
		Plasmid 1 (pAA)^*b*^	94,194	−	+	JBRFVK020000002.1
		Plasmid 2	5,569	−	−	JBRFVK020000003.1
		Plasmid 3	4,076	−	−	JBRFVK020000004.1
		Plasmid 4	2,311	−	−	JBRFVK020000005.1
		Plasmid 5	2,140	−	−	JBRFVK020000006.1

^*a*^ EAEC, enteroaggregative *E. coli*; aEPEC, atypical enteropathogenic *E. coli*.

^*b*^ pAA, plasmid of aggregative adherence.

^*c*^ As shown in Table S3. (+) indicates the presence and (−) indicates the absence of the *eae* and *aggR* genes.

**TABLE 3:** Overview of the pAA plasmids found in the two EAEC and in the hybrid aEPEC/EAEC strains of serotype O3:H2

Pathotype^*a*^	*E. coli* strains	Size (base pairs)	Coding sequences	Plasmid replicon(s)
EAEC	IAL7250	95,100	109	IncFIB/IncFII
	IAL7255	84,405	98	IncFIB/IncFII
aEPEC/EAEC	IAL7252	94,194	107	IncFIB/IncFII

^*a*^ EAEC, enteroaggregative *E. coli*, aEPEC, atypical enteropathogenic *E. coli*.

### *E. coli* of serotype O3:H2 strains belonging to the ST8087 share a similar gene content and display a syntenic chromosomal structure

**Fig 2:**
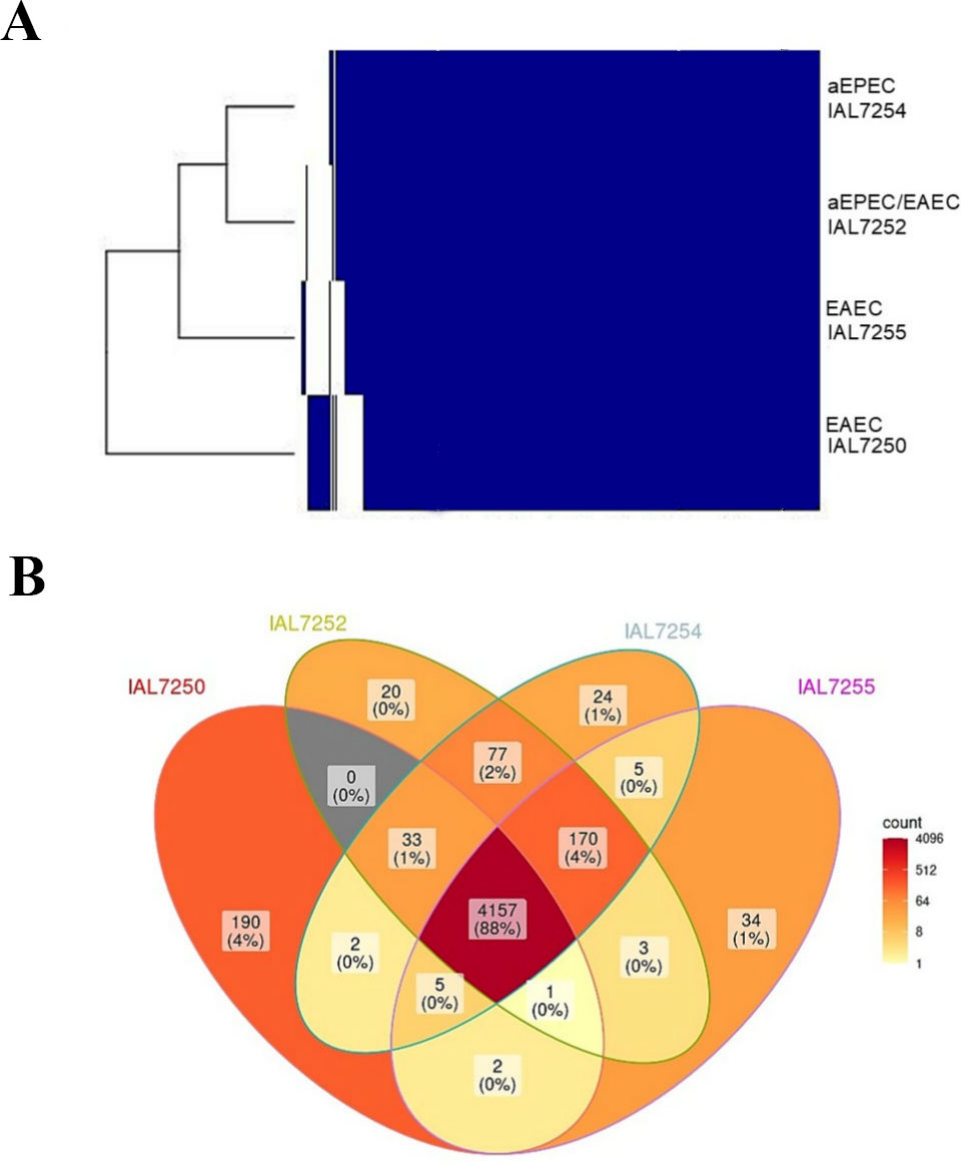
Chromosomal pan- and core-genome analysis of the *E. coli* strains of serotype O3:H2. (**A**) This analysis illustrates the presence (blue) or absence (white) of gene clusters (vertical axis) across the four genomes examined (horizontal axis). Furthermore, hierarchical clustering groups the *E. coli* O3:H2 strains based on their gene-content similarity, showing that the hybrid aEPEC/EAEC is more closely related to aEPEC than to the two EAEC strains analyzed. The pan genome consists of 4,723 gene clusters, of which 4,157 (88.0%) are part of the core genome. (**B**) Venn diagram illustrating the number of chromosomal gene clusters shared among the distinct *E. coli* strains.

To investigate the chromosomal gene content of the 4 representative *E. coli* strains sequenced, we performed pan- and core-genome analyses. The pan-genome comprised 4,723 gene clusters, of which 4,157 (88.0%) were part of the core genome (Table S6; Data S1). Of note, a gene cluster is defined as a group of orthologous genes shared across multiple genomes, based on the sequence similarity and genomic position. Hierarchical clustering revealed that the chromosomal gene content of the hybrid aEPEC/EAEC strain IAL7252 is more similar to that of aEPEC IAL7254 than to that of the EAEC strains IAL7250 and IAL7255 (Fig. 2A). To visually represent the number of genes shared among the *E. coli* strains studied, a Venn diagram was constructed (Fig. 2B). Comparative analysis of the 566 chromosomal gene clusters differentially present among the 4 sequenced *E. coli* of serotype O3:H2 strains revealed a clear separation into 2 major groups: 170 gene clusters were exclusively shared by the aEPEC IAL7254, EAEC IAL7255, and hybrid aEPEC/EAEC IAL7252 strains, all belonging to ST8087, and 190 gene clusters were unique to the EAEC IAL7250, which belongs to ST10 (Fig. 2B; Tables S7 and S8). Furthermore, the aEPEC IAL7254 and hybrid aEPEC/EAEC IAL7252 strains share a set of 77 gene clusters that are absent in the 2 sequenced EAEC strains (Fig. 2B; Table S9).

**Fig 3:**
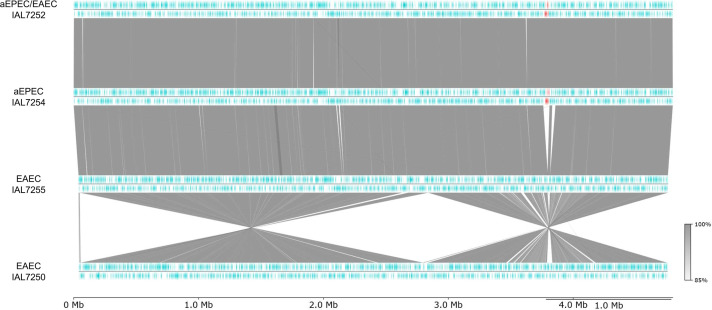
Chromosomal genetic map of the four representative *E. coli* strains of serotype O3:H2. This map illustrates the gene organization on both the forward and reverse strands of the chromosomes of the four representative *E. coli* strains analyzed. Genomic similarity is represented by gray linking bars. Notably, the hybrid aEPEC/EAEC IAL7252, aEPEC IAL7254, and EAEC IAL7255 strains exhibit a syntenic chromosome. The chromosomes of the hybrid aEPEC/EAEC IAL7252 and aEPEC IAL7254 differ from that of EAEC IAL7255, primarily due to the presence of the LEE region, highlighted in red. In contrast, two large chromosomal inversions were detected in the EAEC IAL7250 genome relative to the other three strains.

The chromosomal gene content map of the four representative *E. coli* of serotype O3:H2 strains sequenced revealed that the chromosomes of hybrid aEPEC/EAEC IAL7252, aEPEC IAL7254, and EAEC IAL7255 (all belonging to the ST8087) are syntenic. Notably, the chromosomes of the aEPEC IAL7254 and hybrid aEPEC/EAEC IAL7252 strains differ from that of EAEC IAL7255 mainly due to the presence of the LEE region in the former two strains. In addition, two large chromosomal inversions were identified in EAEC IAL7250 when compared with the other three strains (Fig. 3).

### Identification of chromosomal hotspot regions harboring genes encoding virulence factors in the hybrid aEPEC/EAEC strain

**Fig 4:**
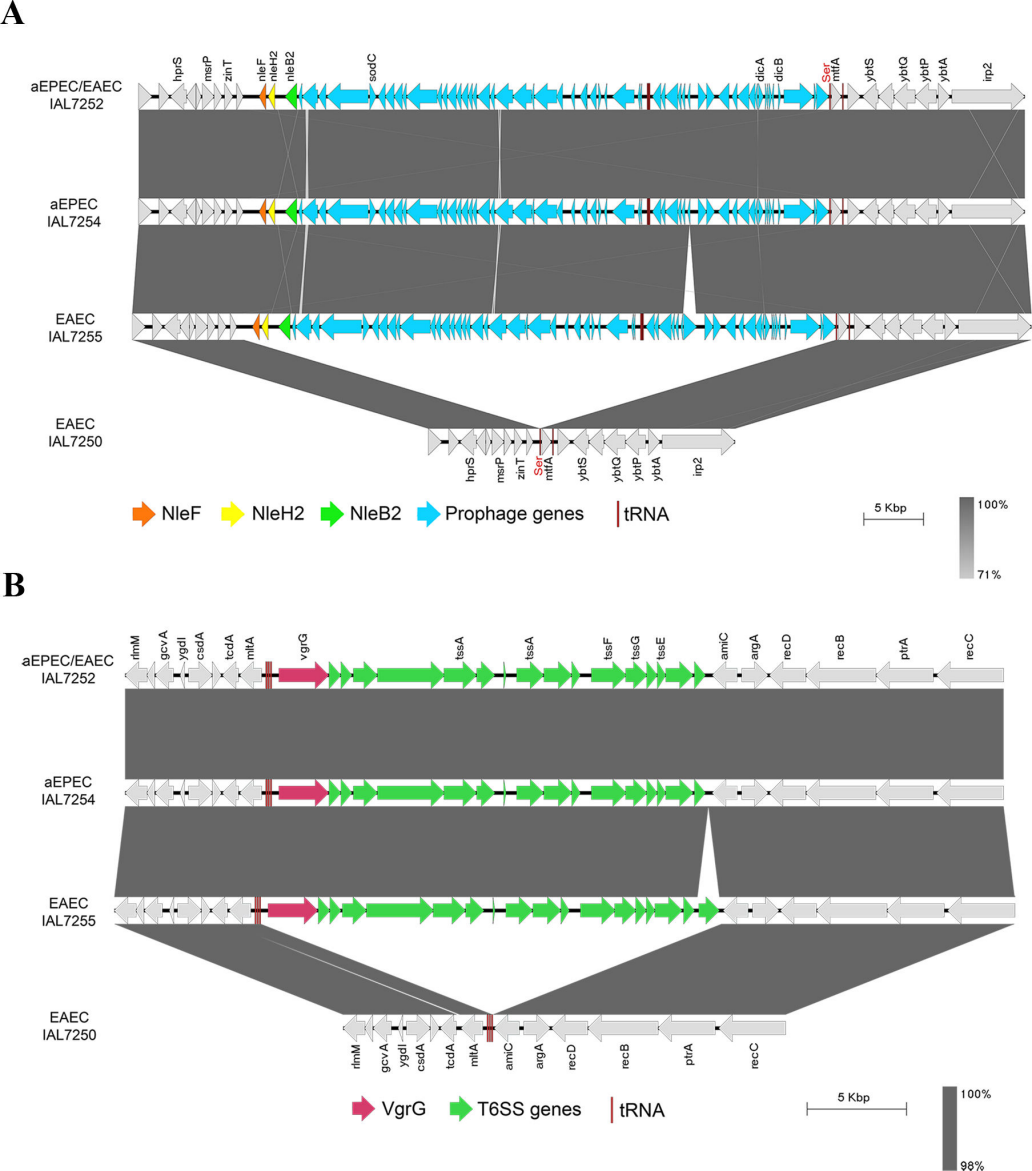
Identification of two chromosomal regions present in the strains from the serotype O3:H2 and ST8087. (**A**) A chromosomal prophage harboring the *nleB2*, *nleF*, and *nleH2* genes, with approximately 47.8 Kb and comprising 58 predicted CDSs, is inserted downstream of the DNA sequence encoding the tRNA for the amino acid serine. (**B**) A chromosomal region containing genes encoding a putative type VI secretion system (T6SS), with approximately 22.0 Kb in length and comprising 17 CDSs, is inserted downstream of the DNA sequence encoding the tRNA for the amino acid methionine.

A detailed analysis of the 170 chromosomal gene clusters exclusively identified in strains from the ST8087 studied (aEPEC IAL7254, EAEC IAL7255, and the hybrid aEPEC/EAEC IAL7252) led to the finding of a chromosomal region containing three genes encoding non-LEE effectors: *nleB2*, *nleF*, and *nleH2* (Table S7). Since these genes are absent in EAEC IAL7250 (ST10), this strain was used as a reference to determine both the size and the insertion site of this region in the chromosome of the hybrid aEPEC/EAEC IAL7252 strain. This analysis revealed that this region spans approximately 47.8 Kb, comprises 58 coding sequences (CDSs), and is inserted downstream of the sequence encoding the transfer RNA (tRNA) for the amino acid serine (Fig. 4A; Table S10).

The annotation of the genes present in this 47.8 Kb chromosomal region (Table S10) suggests that it may represent a prophage. To test this hypothesis, we employed the PHASTEST online tool to detect prophage regions integrated into the chromosome of the hybrid aEPEC/EAEC IAL7252 strain. This analysis identified 1 incomplete prophage (region 2) and 5 intact prophages (regions 1, 3, 4, 5, and 6). Importantly, the genes *nleB2*, *nleF*, and *nleH2* were found within prophage region 6 (Table S11), confirming our initial hypothesis. Moreover, all 6 prophages were also detected in the aEPEC IAL7254 and EAEC IAL7255 strains, whereas the EAEC IAL7250 strain contains only prophages 2, 3, 4, and 5, as shown in Fig. S1.

In addition, we identified another chromosomal region of approximately 21.5 Kb, containing genes predicted to encode a putative type VI secretion system (T6SS). This region comprises 17 CDSs and is inserted downstream to the sequence encoding the tRNA for the amino acid methionine (Fig. 4B; Table S12).

Among the 77 chromosomal gene clusters exclusively found in the aEPEC IAL7254 and hybrid aEPEC/EAEC IAL7252 strains (Fig. 2B; Table S9), we highlight the 41 genes belonging to the LEE region (approximately 34.2 Kb). An online tool that classifies the LEE region into 30 distinct subtypes revealed that both aEPEC IAL7254 and hybrid aEPEC/EAEC IAL7252 carry the LEE region of subtype 8, exhibiting 100% nucleotide sequence identity and 100% coverage (data not shown).

**Fig 5:**
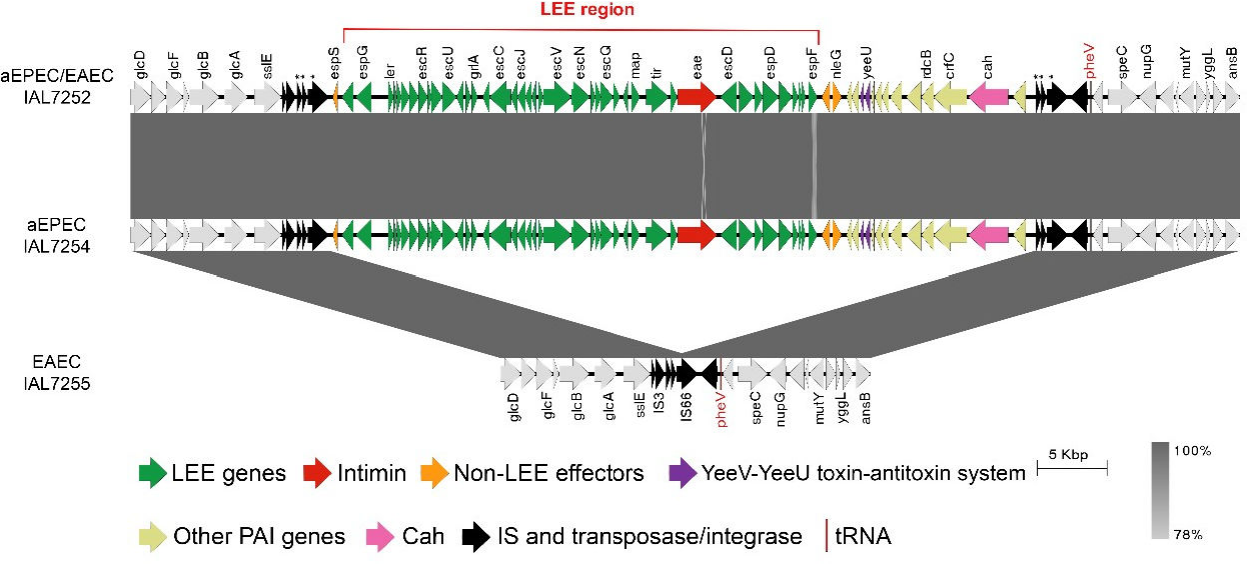
The aEPEC IAL7254 and hybrid aEPEC/EAEC IAL7252 strains harbor the LEE region of subtype 8. The LEE-containing region is approximately 49.8 Kb in length, comprising 57 CDSs, and is inserted downstream of the tRNA *pheV*. Notably, the LEE region (~34.2 Kb organized into 41 CDSs) in both the aEPEC IAL7254 and hybrid aEPEC/EAEC IAL7252 strains is subtype 8. The LEE region is flanked on the right by the *espS* pseudogene and the left by 15 genes, including those encoding two non-LEE effectors (EspM and NleG), the YeeV-YeeU toxin-antitoxin system, and the autotransporter adhesin Cah. Asterisks (*) indicate DNA sequences repeated at the 5′ and 3′ ends.

Using the EAEC IAL7255 strain, which lacks the LEE region, as a reference, we observed that the LEE region is part of a chromosomal PAI of approximately 49.8 Kb, comprising 57 CDSs and inserted downstream of the tRNA *pheV*. At both the 3′ and 5′ ends of the LEE-containing region, we identified several integrative elements, including insertion sequences (IS), along with genes encoding transposases and integrases, which are also present at the same locus in the chromosome of the EAEC IAL7255 strain that lacks the LEE region. In addition, we observed that three of these integrative elements are repeated at both ends of the LEE-containing region in the hybrid aEPEC/EAEC IAL7252 strain (Fig. 5; Table S13).

Expanding the analysis of the LEE region to its 3′ and 5′ flanking regions, we observed the presence of an *espS* pseudogene on the left and genes encoding the non-LEE effectors EspM and NleG, the YeeV-YeeU toxin-antitoxin system, and the Cah autotransporter adhesin on the right (Fig. 5; Table S13).

### High genetic conservation of the pAA plasmid in EAEC and hybrid aEPEC/EAEC strains of serotype O3:H2

**Fig 6:**
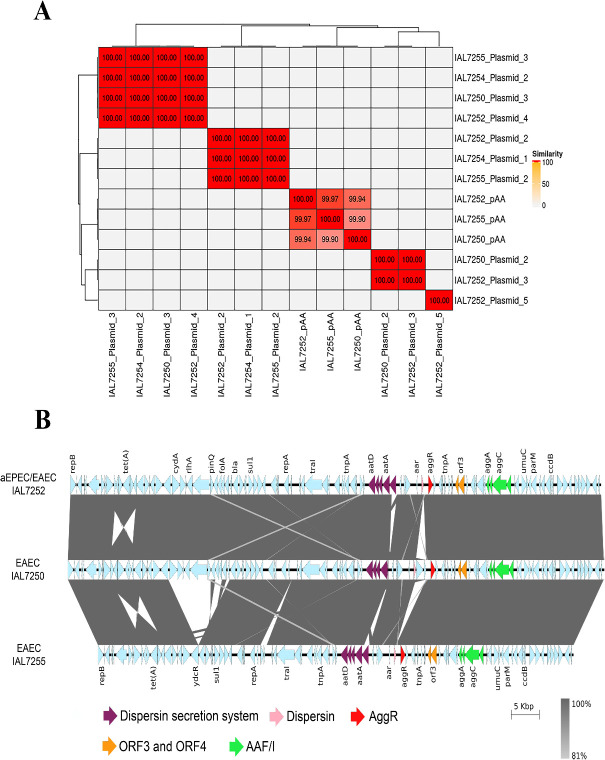
Genomic comparison of the plasmids present in the *E. coli* strains of the serotype O3:H2 studied. (**A**) Comparison of the nucleotide sequence of the identified plasmids revealed five distinct plasmid types, with the pAA found in EAEC (IAL7250 and IAL7255) and aEPEC/EAEC hybrid (IAL7252) strains sharing approximately 100% identity. Only alignment identities with more than 50% coverage were considered for generating this image (Table S14). As observed for the pAA plasmid, cryptic plasmids are also shared among the studied *E. coli* O3:H2 strains, suggesting vertical inheritance and co-evolution with their host lineages. (**B**) In this image, gray connecting bars indicate shared genomic regions among the plasmids. The six-gene block containing the *tetA* gene is conserved across all plasmids but appears inverted in the plasmid from strain IAL7250 relative to those from strains IAL7252 and IAL7255. The overlapping regions observed in this area result from identical flanking genes and represent alternative possible arrangements generated by recombination events. Note that all three pAA plasmids harbor key genes encoding virulence factors associated with EAEC pathogenesis, such as ORF3, ORF4, *aggR*, and *aap*, as well as the *aggDCBA* operon. Notably, the *aatP* gene in the EAEC IAL7250 strain contains a 978-bp deletion at its 5′ end.

A comparison of the nucleotide sequences among the plasmids identified in the sequenced *E. coli* strains of serotype O3:H2 revealed five distinct plasmid types, including pAA, which were differentially distributed across the four representative strains (Fig. 6A). All identity and coverage percentages observed for the nucleotide sequences of the plasmids identified are provided in Table S14. Additionally, genes encoding virulence factors and antimicrobial resistance were exclusively found in the pAA plasmid (Tables S4 and S5).

A comparative analysis of the 3 pAA plasmids found in the two EAEC (IAL7250 and IAL7255) and hybrid aEPEC/EAEC IAL7252 strains revealed that these plasmids are highly similar in terms of length (Kb), as well as in the number and composition of CDSs. Additionally, all 3 pAA plasmids simultaneously carried the IncFIB and IncFII replicons (Table 3). Sequence comparison of the 3 sequenced pAA plasmids showed approximately 100% identity with at least 85.7% coverage (Fig. 6A; Table S14).

Several genes encoding key virulence factors associated with the EAEC pathogenesis were identified in the 3 pAA plasmids analyzed, including ORF3, ORF4, *aggR*, *aap*, as well as the *aggDCBA* operon (Fig. 6B). In the hybrid aEPEC/EAEC IAL7252 and EAEC IAL7255 strains, the complete *aatPABCD* operon was detected, whereas in the EAEC IAL7250, the *aatP* gene exhibited a 978 bp deletion in its 5′ region, although the other genes from this operon were intact. Furthermore, the 3 pAA plasmids also carry genes encoding proteins mediating antimicrobial resistance, such as *bla_TEM_*_-1_, *tet(B)*, *aph(6)-Id*, *aph(3'')-Ib*, and *sul2*. The *dfrA8* gene was found in the pAA plasmids of the EAEC IAL7250 and hybrid aEPEC/EAEC IAL7252 strains, but not in the pAA of EAEC IAL7255 (Table S5).

## DISCUSSION

The plasticity of the *E. coli* genome has facilitated the emergence of strains carrying genetic markers from two or more DEC pathotypes, which are currently classified as hybrids (28, 32). Hybrid EPEC/EAEC are defined as *E. coli* strains that simultaneously harbor the chromosomal LEE region and the pAA plasmid, which are genetic markers of the EPEC and EAEC pathotypes, respectively (28). Although rare, such hybrids have been isolated from individuals with diarrhea in Brazil, with these strains belonging to serotypes O142:H34, O158:HNM, and O3:H2 (33–35), as well as in studies conducted in Mexico and Colombia (36–38). It is important to note that most hybrid EPEC/EAEC strains identified to date lack genetic markers from the pEAF plasmid, such as the genes of the *bfp* operon, and are therefore classified as aEPEC/EAEC (28).

Notably, hybrid aEPEC/EAEC strains of serotype O3:H2 were recovered during a diarrheal outbreak investigation in Brazil, in which both aEPEC and EAEC strains of the same serotype were also identified (33). The detection of *E. coli* strains of the serotype O3:H2 with distinct virulence gene profiles during the same outbreak investigation may suggest that the bacterial population present at the time was closely interacting, likely within the gastrointestinal tracts of the affected individuals, facilitating horizontal gene transfer among co-circulating *E. coli* strains. In line with this notion, the fact that the *E. coli* strains of serotype O3:H2 and ST8087 studied are phylogenetically related, exhibit a highly similar gene content, and share key chromosomal genetic elements supports the hypothesis that these strains originated from a common ancestor, which, through multiple horizontal gene transfer events, gave rise to the aEPEC, EAEC, and hybrid aEPEC/EAEC pathogenic groups.

A study published in 2016 proposed the classification of the LEE region into 30 distinct subtypes (39). Using this classification, we identified that the aEPEC IAL7254 and hybrid aEPEC/EAEC IAL7252 strains, analyzed in the present study, harbored an LEE region of subtype 8. This subtype has previously been detected in *E. coli* strains belonging to the following STs: 3, 20, 21, 29, 40, and 328 (39). The aEPEC strains from these STs belong to the phylogenomic lineages EPEC2, EPEC7, EPEC12, and EHEC2, all of which are part of phylogroup B1 (40). To the best of our knowledge, this is the first report of the LEE region of subtype 8 in *E. coli* strains from phylogroup A, supporting prior evidence that the LEE region is a mobile genetic element capable of horizontal transfer between distinct bacterial genomes (11, 41).

The analysis of the LEE flanking regions in several sequenced EPEC strains has demonstrated that the genetic elements present in the 5′ and 3′ flanking regions differ considerably among distinct strains (42). Examples of genetic elements integrated into the flanking regions include IS, prophages, the *efa1/lifA* region (harboring the *efa1/lifA*, *nleB*, *nleE*, and *espL2* genes), and non-LEE-encoding genes (e.g., *ibe*, *espM*, and *nleG*) (43–47).

An example of an *E. coli* strain whose genome has been fully resolved is the tEPEC prototype B171 (44), which belongs to phylogroup B1 and harbors an LEE region of subtype 8 (39). Comparison of the LEE region of subtype 8, as well as its flanking sequences, from tEPEC B171 (phylogroup B1) with those identified in the aEPEC IAL7254 and hybrid aEPEC/EAEC IAL7252 (both from the phylogroup A) strains revealed that these genomic elements are almost identical both in the core and in the flanking regions (≥99% of identity with 100% coverage, data not shown). Curiously, the terminal portion of the right flanking region of the LEE subtype 8 is nearly identical (≥90% of identity with ≥90% coverage) to a part of the Sakai prophage-like element 1 (SpLE1) (44). SpLE1 is a large integrative element identified in the chromosome of the *E. coli* O157:H7 Sakai prototype strain, which harbors genes encoding a type IV toxin-antitoxin system and the autotransporter adhesin Cah (48). The presence of part of the SpLE1 element integrated alongside the core of the LEE region may suggest that the LEE region of subtype 8 has undergone a complex evolutionary history, ultimately resulting in the genetic structure observed today. Based on these findings, we hypothesize that an EPEC strain from phylogroup B1 may have transferred the LEE region—of subtype 8—to an *E. coli* strain of serotype O3:H2 and ST8087, contributing to the emergence of *E. coli* strains from the phylogroup A harboring this LEE subtype, such as the strains analyzed in the present study.

All *E. coli* strains from the serotype O3:H2 and ST8087 analyzed in this study were collected in the same year, from the same geographic region, and during a diarrhea outbreak investigation (33). This context, combined with the lack of a significant number of other sequenced *E. coli* strains from this ST in public databases, represents a limitation of our study. Consequently, elucidating the sequence in which the distinct mobile genetic elements were acquired, and, in turn, determining which pathogenic group emerged first, remains a complex and unresolved question.

Furthermore, another limitation of this study is that we did not investigate the impact of the presence or absence of important mobile genetic elements (including the LEE region, the pAA plasmid, a locus encoding a putative T6SS, or a prophage carrying the *nleB2*, *nleF*, and *nleH2* genes) or the effect of the differences in gene organization observed between strains from ST8087 and ST10 on the virulence of these pathogens. Based on the available evidence, we can hypothesize that genetic elements found in the studied strains may significantly influence both the ability of the strains to cause diarrhea and the severity of the disease, mainly because they carry key virulence factor-encoding genes. To test this hypothesis, ongoing studies in our laboratory are focused on elucidating the impact of these genetic variations on bacterial pathogenicity.

It is important to note that the differences in gene organization observed between ST8087 and ST10 strains may represent genuine biological rearrangements resulting from evolutionary events such as recombination or inversion. However, we cannot completely rule out the possibility that some of these differences arise from technical limitations associated with sequencing or genome assembly. Although the assemblies were generated using hybrid approaches that integrate both short- and long-read data, with deep coverage and current assembly software, which supports a high level of confidence in sequence contiguity, assembly-related artifacts may still occur. Therefore, the observed rearrangements should be interpreted with appropriate caution. In conclusion, our findings suggest that all *E. coli* strains of serotype O3:H2 and ST8087 studied likely originated from a common ancestor, which, through multiple horizontal gene transfer events, contributed to the emergence of the aEPEC and EAEC pathotypes, as well as the hybrid aEPEC/EAEC strain.

## MATERIALS AND METHODS

### *E. coli* strains of serotype O3:H2 studied

In the present study, we evaluated eight *E. coli* strains of serotype O3:H2 obtained during a diarrheal outbreak investigation in Brazil (33). Of note, these strains belonged to the following pathotypes: aEPEC (IAL7254), EAEC (IAL7250, IAL7251, IAL7255, and IAL7257), and hybrid aEPEC/EAEC (IAL7252, IAL7253, and IAL7256).

### DNA extraction, genome sequencing, assembly, and annotation

Genomic DNA of the *E. coli* strains of serotype O3:H2 was extracted from overnight cultures in lysogeny broth (LB) incubated at 37°C with shaking at 200 rpm, using the QIAmp DNA extraction kit (QIAGen, Germany).

The genomes of the IAL7254, IAL7253, and IAL7256 were sequenced using 2 × 250 paired-end read libraries on the Illumina HiSeq 1500 at Butantan Institute, performed according to the manufacturer’s protocol for sequencing. The IAL7250, IAL7251, IAL7252, IAL7255, and IAL7257 strains were sequenced by the MicrobesNG sequencing service (Birmingham Research Park, Birmingham, England) following protocols of the 2 × 250 Illumina paired-end short reads. To remove adapters, low-quality sequences, and small fragments from the raw reads, we applied the FastP (v0.23.2) software (https://github.com/OpenGene/fastp) (49). In addition, to remove possible human genome contamination sequences, we used the Bowtie2 (v2.5.0) software (http://bowtie-bio.sourceforge.net/bowtie2/index.shtml) (50). Prior to assembly, the sequenced raw reads were quality-checked by FastQC (v0.12.0) (https://www.bioinformatics.babraham.ac.uk/projects/fastqc/) (51) and MultiQC (v1.13) (https://multiqc.info/) (52) software. The Illumina short reads were assembled using SPAdes (v3.15.5) (53). Contigs smaller than 200 bp were discarded by BBmap (v39.27) (https://jgi.doe.gov/search?search_api_fulltext=data%20tools%20bbtools).

Four representative *E. coli* strains of serotype O3:H2 (aEPEC IAL7254, EAEC IAL7250, EAEC IAL7255, and hybrid aEPEC/EAEC IAL7252) were long-read-sequenced using the Nanopore Technologies Oxford (ONT) MinION, following the Rapid Barcoding kit protocol (SQK-RBK004, ONT, Oxford, England) with a MinION R9.4 flow cell (ONT, Oxford, England). Base calling was performed using Guppy software (v5.1, ONT). The Unicycler (v0.5.0) (https://github.com/rrwick/Unicycler) (54) program was used to generate the hybrid assembly combining short and long reads. Further, the hybrid DNA assembly was polished using Medaka (v1.8.1) software (ONT, https://github.com/nanoporetech/medaka) and Polypolish (v0.5.0) (https://github.com/rrwick/Polypolish) (55).

Gene annotation was done using Prokka (v1.14.5) software (https://github.com/tseemann/prokka) (56).

### Molecular typing of the eight *E. coli* genomes sequenced

First, the *in silico* serotype of each of the eight *E. coli* strains studied was confirmed using the online website Serotype Finder (v2.0.1) (https://cge.food.dtu.dk/services/SerotypeFinder/) (57) with default settings. Then, the eight *E. coli* strains were assigned into one of the distinct *E. coli* phylogroups, using the ClermonTyping (v23.06) (http://clermontyping.iame-research.center/) (58), and the ST was determined using the multilocus sequence typing (MLST) analyses (59) in the MLST (v2.0.9) software (https://github.com/tseemann/mlst) (60), which incorporates the PubMLST (61).

Genes encoding virulence factors and antimicrobial resistance were identified using the online tools VirulenceFinder (v2.0.5) (https://cge.food.dtu.dk/services/VirulenceFinder/) (62–64) and ResFinder (v4.6.0) (http://genepi.food.dtu.dk/resfinder) (62, 65) respectively.

### SNP-based phylogenetic analyses

A SNP-based phylogenetic tree was generated using the genomes of the eight outbreak-associated *E. coli* strains of serotype O3:H2 sequenced herein, 72 aEPEC, and 84 EAEC strains, all belonging to phylogroup A, 37 *E. coli* genomes representing the distinct phylogroups (A, B1, B2, C, D, E, F, and G), together with the genome of *E. fergusonii* ATCC 35469, used as an outgroup (Table S15). A maximum-likelihood phylogenetic tree was constructed using the kSNP4.0 software (66), based on all SNPs identified among the genomes analyzed. FastTree2, integrated into kSNP4.0, was used to infer the phylogenetic tree and assess branch support values using the Shimodaira–Hasegawa test with 1,000 resamples (67). The resulting phylogenetic tree was visualized using the online tool iTOL (https://itol.embl.de/) (68).

### Plasmids nucleotide sequence comparison and replicon(s) identification

To compare the percentage of nucleotide identity among the plasmids found in the four representative *E. coli* strains of serotype O3:H2 studied, we used the Average Nucleotide Identity (ANI) (v0.2.12) software (https://github.com/widdowquinn/pyani) (69) with default settings and BLAST as an alignment option. Plasmid replicons were identified using the online platform PlasmidFinder (v2.0.1) found at https://cge.food.dtu.dk/services/PlasmidFinder/ (62, 70).

### Chromosomal pan- and core-genome analysis

The chromosomal pan- and core-genome analysis of the four representative *E. coli* strains selected was performed using the Roary software (v3.13.0) (https://github.com/sanger-pathogens/Roary) (71). To better visualize the gene clusters unique or shared among the four *E. coli* strains analyzed, a Venn diagram was constructed using the R environment (72).

To refine the gene annotation provided by Roary (71), we conducted a BLASTN (62) analysis utilizing DEC prototypes B171 (GenBank: ASM16789v4), E2348/69 (NC_011601), 041-2 (GenBank: GCA_000027125.1), 17-2 (GenBank: NZ_JACEFV000000000.1), CFT073 (NZ_CP058222.1), and K12 MG1655 (NZ_CP097883.1). Briefly, genome annotations obtained from NCBI were used to replace Roary-generated annotations for gene clusters classified as “group.” The resulting data were systematically processed and visualized within the R environment (72).

### Prophage identification

The PHASTEST software (v3.0) (https://phastest.ca/) (73–75) was used to identify prophage regions present in the chromosome of the hybrid aEPEC/EAEC IAL7252 strain.

### LEE subtyping

The LEE region subtyping from the aEPEC IAL7254 and hybrid aEPEC/EAEC IAL7252 strains was performed using the SRST2 software (v0.2.0) (https://github.com/katholt/srst2) (76). Of note, this software allowed the differentiation of the LEE region into 30 distinct subtypes, as previously described (39).

### DNA sequence comparison using Easyfig

Easyfig (v3.0.0) was employed to illustrate the DNA sequence comparison from the representative *E. coli* strains included in this study (https://mjsull.github.io/Easyfig/) (77).

### Chromosomal gene map construction

Genome alignments were generated using pyGenomeViz (v1.6.0) (https://github.com/moshi4/pyGenomeViz) (78), with MUMmer (79) as the embedded alignment tool.

## Data Availability

The GenBank accession numbers for the genome assemblies generated in the present study are listed in Table 2 and Table S1 and can be accessed at https://www.ncbi.nlm.nih.gov/. Furthermore, all FASTA and GBK files used in the analyses presented in this study are available at https://doi.org/10.6084/m9.figshare.30266353.

## SUPPLEMENTAL MATERIAL

Data S1 spectrum.02774-25-s0001.txt Nucleotide sequence in FASTA format of the chromosomal gene clusters identified in the pan- and core-genome analysis performed with the four representative *E. coli* strains of serotype O3:H2 sequenced.

Figure S1 spectrum.02774-25-s0002.pdf Chromosomal localization of prophage regions identified in the hybrid aEPEC/EAEC IAL7252 strain and in other *E. coli* O3:H2 genomes analyzed in this study.

Supplemental tables spectrum.02774-25-s0003.xlsx Tables S1 to S16.

ASM does not own the copyrights to Supplemental Material that may be linked to, or accessed through, an article. The authors have granted ASM a non-exclusive, world-wide license to publish the Supplemental Material files. Please contact the corresponding author directly for reuse.
