# Supplementary material for: Genome on the move: emergence of hybrid atypical enteropathogenic/enteroaggregative Escherichia coli (aEPEC/EAEC) during a diarrheal outbreak in Brazil
Source: Microbiol Spectr. 2026 Apr 3;14(5):e02774-25. doi: 10.1128/spectrum.02774-25 (PMC13141912; doi:10.1128/spectrum.02774-25)
Supplement: Figure S1 — Chromosomal localization of prophage regions identified in the hybrid aEPEC/EAEC IAL7252 strain and in other E. coli O3:H2 genomes analyzed in this study. [file spectrum.02774-25-s0002.pdf]

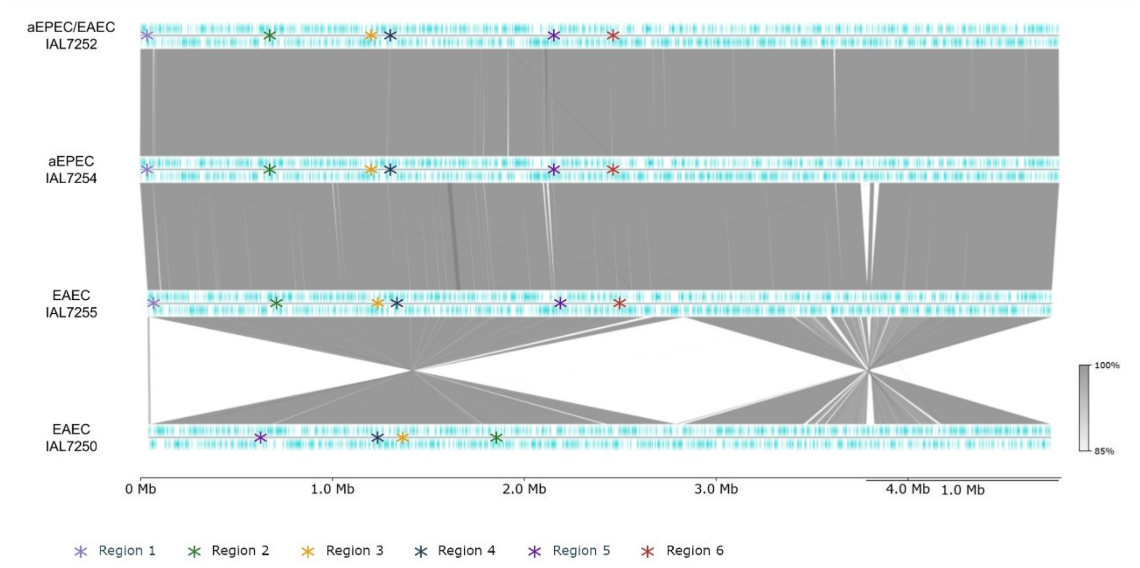

Figure S1. Chromosomal localization of prophage regions identified in the hybrid aEPEC/EAEC IAL7252 strain and in other *E. coli* O3:H2 genomes analyzed in this study. This chromosomal genetic map shows the gene organization on the forward and reverse strands of the chromosomes of four representative *E. coli* O3:H2 strains. The six prophage regions identified in the hybrid IAL7252 are indicated by asterisks (*), and each prophage is distinguished by a unique color. Notably, all six prophage regions identified in the hybrid aEPEC/EAEC IAL7252 strain are also present in aEPEC IAL7254 and EAEC IAL7255, both belonging to ST10, whereas EAEC IAL7250 (ST8087) lacks prophage regions 1 and 6. Genomic similarity among the strains is indicated by gray connecting bars.
